# Characterization of ultramarine blue in Roman wall paintings: case study from Volsinii (Bolsena, Italy)

**DOI:** 10.1007/s00216-025-05745-y

**Published:** 2025-02-08

**Authors:** Martina Bernabale, Anna Candida Felici, Pierfrancesco Atanasio, Luca Buccini, Daniele Passeri, Marco Rossi, Paolo Binaco, Danilo Dini

**Affiliations:** 1 Department of Basic and Applied Sciences for Engineering (SBAI), Sapienza University of Rome, Via Antonio Scarpa 14, 00161 Rome, Italy; 2 Centro Di Ricerca Per Le Nanotecnologie Applicate All’Ingegneria Della Sapienza (CNIS), Sapienza University of Rome, Piazzale A. Moro 5, 00185 Rome, Italy; 3 Museo Territoriale del Lago Di Bolsena, Piazza Monaldeschi 1, 01023 Bolsena, VT Italy; 4 Department of Chemistry, Sapienza University of Rome, Piazzale Aldo Moro 5, 00185 Rome, Italy

**Keywords:** ED-XRF, Micro-Raman spectroscopy, XRPD, Ultramarine blue, Volsinii

## Abstract

The identification of blue pigments in Roman wall paintings provides crucial insights into the materials, artistic techniques, and trade routes in the antiquity. The present study focuses on the analysis of a blue pigment lump discovered at Ponticello (Bolsena, Italy) initially thought to be Egyptian blue. The lump pigment is compared with two synthetic ultramarine pigments (Poggi and Divolo), “Herculaneum blue,” Egyptian blue, and natural lapis lazuli from Monte Somma, Italy. Through a multi-analytical approach that comprehends energy-dispersive X-ray fluorescence (ED-XRF) spectroscopy, micro-Raman spectroscopy, and X-ray powder diffraction (XRPD), the pigment was univocally identified as natural lazurite, the primary component of lapis lazuli. Chemometrics, including principal component analysis (PCA), was applied to the ED-XRF data to reveal patterns in the elemental composition of the samples. This finding is significant as ultramarine blue was a rare and expensive material in Roman times, which was traditionally sourced from distant regions like Afghanistan. While this study does not aim to determine the geographical provenance of the pigment, it offers important insights into Roman artistic practices, economic resources, and trade networks. Future research will explore the chemical composition of the pigments present in the frescoes from the archaeological area of Volsinii to provide important further insights into ancient material exchanges.

**Graphical Abstract:**

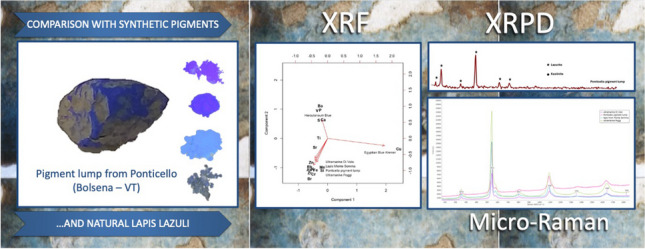

**Supplementary Information:**

The online version contains supplementary material available at 10.1007/s00216-025-05745-y.

## Introduction

Identifying pigments in wall paintings is essential for a better understanding of the raw materials and techniques employed, in order to ensure an effective preservation of this type of painting [1, 2]. Such an information is, in fact, crucial for selecting and implementing the most appropriate conservation and restoration methods [3]. In archaeology, the development of archaeometry associated with the availability of advanced analytical and spectroscopic techniques is of great help in unrevealing the aspects related to materials, to pictorial/construction techniques, and to the direction of the exchanges of goods, people, skills, and ideas in the antiquity. The current studies on the wall paintings/frescoes of the Roman Era still show lacunas about the use of blue pigments, due to the rarity and the higher cost compared to the more commonly used red and yellow [4–7].

According to the writers Pliny and Vitruvius, blue pigments in Roman wall paintings were made using materials corresponding to azurite [Cu(CO_3_)]_2_Cu(OH)_2_, lapis lazuli (Na,Ca)_8_Al_6_Si_6_O_24_(S,SO_4_^2−^)_2_, indigo(C_16_H_10_N_2_O_2_), and Egyptian blue (CaCuSi_4_O_10_) [4]. Indigo, obtained from the plant *Indigofera anil*, was mostly used for dyeing textiles and is rarely found in wall paintings [8]. In contrast, Egyptian blue, also known as cuprorivaite, was extensively used as a standard blue pigment in Roman wall paintings [9, 10]. During the Roman period, Egyptian blue was commonly distributed across the Empire in a standardized form of small balls, approximately 15 to 20 mm in diameter. This allowed painters to control the grain size when grinding the pigment, thereby adjusting the shade of blue and its covering power according to their needs [11]. The pigment was produced by heating a mixture of limestone, malachite, and quartz sand to temperatures exceeding 850 °C [12]. It has been noted that ultramarine, obtained naturally from lapis lazuli and known as lazurite, is rarely featured in Roman wall paintings [11]. Actually, only a few documented examples include the Servilia tomb [6], the Roman Villa of Baños de Valdearados (fourth–fifth century) in Burgos (Spain), where lazurite was found mixed with limonite to create a green pigment [13], and a villa in the Roman colony of Colchester (UK), where lazurite was identified as a pure blue pigment [14].

This study aims to determine the nature of the blue discovered at Ponticello, a locality near Bolsena (province Viterbo, Italy) as part of a broader investigation into the pigments used in Roman frescoes at the Volsinii site. The discovery of the blue pigment at Ponticello, initially catalogued as Egyptian blue, offers an opportunity to expand our understanding of the materials used in Roman wall paintings. This research aims also to contribute to the wider goal of studying the composition and sourcing of pigments in the region, particularly those found in frescoes at Volsinii. The main aim of this study is to determine the identity of the pigment lump, which has been recently analyzed through fiber optics reflectance spectroscopy (FORS). This spectroscopic analysis suggests it may consist in ultramarine rather than the previously assumed Egyptian blue [15]. To achieve this, a range of analytical-spectroscopic techniques that include energy-dispersive X-ray fluorescence (ED-XRF) spectroscopy, micro-Raman spectroscopy, and X-ray powder diffraction (XRPD) have been employed to provide a comprehensive chemical and mineralogical characterization of the pigment [16, 17]. Future investigations, focusing on the determination of pigment provenance, will provide valuable insights into potential trade routes and local sources of pigments used in Roman times.

## Material and methods

### Materials and archaeological context

**Fig. 1:**
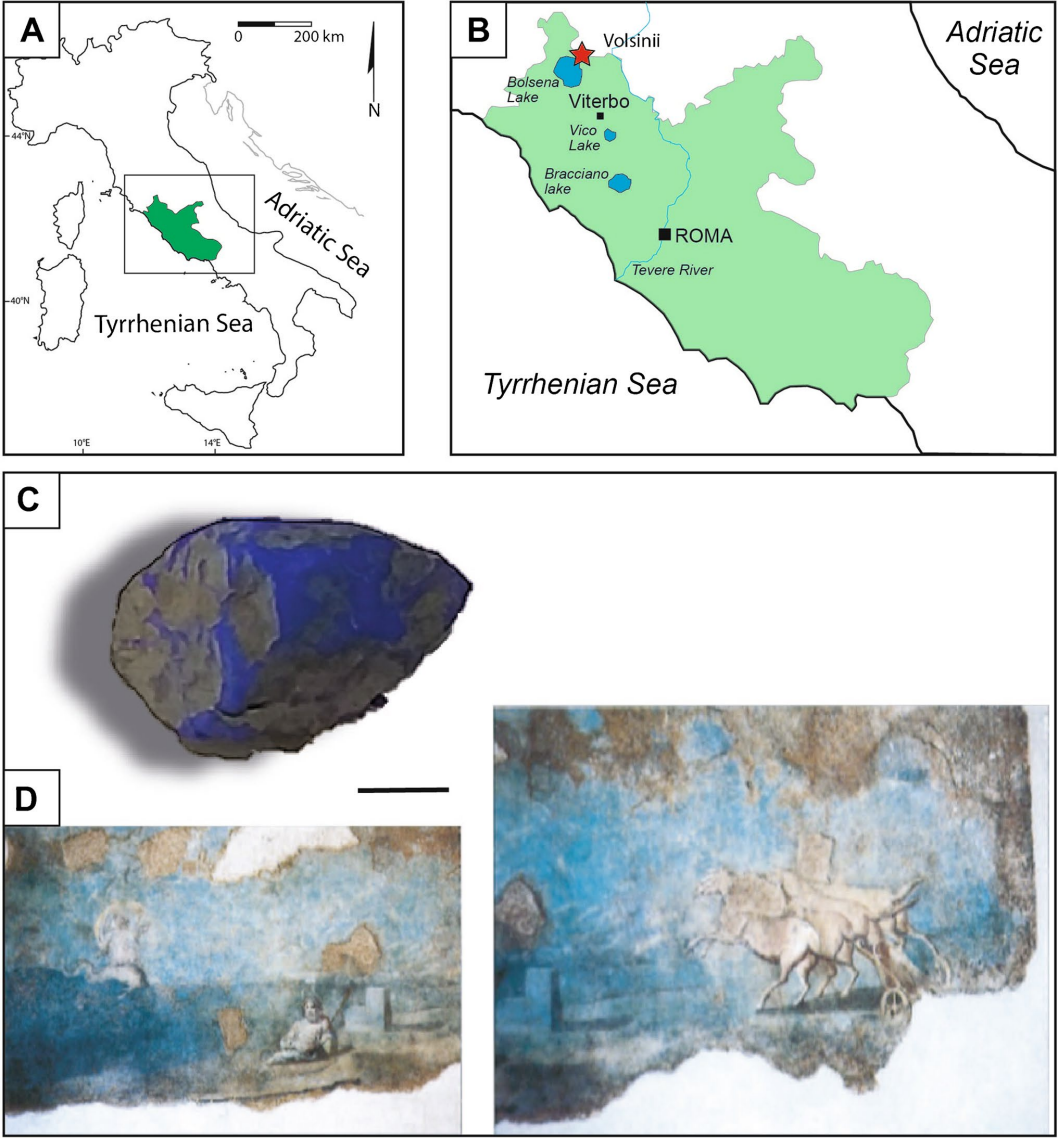
**A** Map of the Lazio region within the Italian Peninsula. **B** Location of the archaeological area of Volsinii (red star). **C** Pigment lump from Ponticello (Bolsena, Viterbo). Scale size, 2 cm. **D** Detail of the fresco from the forensic latrine of Volsinii, currently preserved and exposed at the Museo Territoriale del Lago di Bolsena [modified from Tamburini (1998)]

For the analysis, some fragments of a friable blue pigment collected from the locality of Ponticello, near Bolsena, were studied. Ponticello is situated a few kilometers north-east of the ancient Volsinii, outside the main area of the ancient city. The pigment lump was discovered accidentally during agricultural activities in an area historically associated with Etruscan and Roman settlements, which remains largely unexplored archaeologically. The discovery in the Volsinii area underscores its potential relevance to the artistic practices of the period. Frescoes from the public spaces of the Etruscan-Roman settlement of Volsinii at Poggio Moscini (Bolsena, Viterbo), particularly those preserved at the Museo Territoriale del Lago di Bolsena [18, 19], offer a compelling example of the region’s artistic heritage. While a direct connection between the Ponticello pigment lump and the pigments used in the frescoes of the forica cannot be definitively established, the pigment’s presence in this context sheds light on the materials potentially available to local artists of the time. These murals exhibit elaborate decoration on floors and walls, with the upper walls featuring vine patterns on a white background and a fully blue lower section. On this blue background, mythological and burlesque scenes are depicted, including an empty chariot with prancing horses (possibly referencing the myth of Hippolytus), the titan Ocean as a pot-bellied man holding an oar, a Nereid riding a dolphin, and pygmies in a boat with a donkey-headed prow. This unique decorative scheme dates to approximately 30–40 AD. Among the several colors present in the mural of the forica, particular attention has been dedicated to the analysis of the blue portions of the fresco due to the generally rare occurrence of this color in the decorations dated in the Roman times (Fig. 1). For comparison, two synthetic ultramarine pigments from the manufacturers Poggi and Divolo were analyzed. Additionally, synthetic pigments labeled “Herculaneum blue” and Egyptian blue were also included in the present comparative analysis, along with a powdered lapis lazuli from Monte Somma (Somma-Vesuvius Complex, Naples Province, Campania, Italy). These reference materials, spanning various origins and compositions, were selected to expand the comparative framework and enhance the analysis. The inclusion of Monte Somma lapis lazuli particularly motivated its geographic proximity, as it represents one of the closest known sources of lazurite compared to more distant deposits, such as those in Afghanistan. A summary table of the samples analyzed is provided in Table 1 for clarity and reference.

**Table 1:** Description of the different samples of pigments that have been analyzed in the present study. For the various pigments, the corresponding origin is reported on the first column from right

Sample ID	Description	Origin
Ponticello pigment lump	Friable blue pigment	Ponticello, Bolsena, Lazio, Italy
Ultramarine blue (PA/0561)	Synthetic ultramarine pigment	Poggi manufacturer, Italy
Ultramarine blue (120,069/41)	Synthetic pigment	Divolo manufacturer, Italy
Herculaneum blue (120,074/6042.80)	Synthetic pigment	Abralux manufacturer, Italy
Egyptian blue (10,060)	Synthetic pigment	Kremer manufacturer, Italy
Monte Somma lapis lazuli (7819/2)	Natural powdered sample	Somma-Vesuvius Complex, Naples, Campania, Italy

### X-ray fluorescence spectroscopy

The elemental analyses were carried out by an ED-XRF spectrometer consisting of an X-ray tube (Amptek Mini-X, Amptek Inc., Bedford, UK) with an anode target of Rh and a Be window of thickness 127 μm. The tube was powered with an accelerating potential difference of 40 kV and an electronic current of 80 μA. The detector is a Peltier-cooled Si drift with integrated amplifier and multichannel analyzer (Amptek 123-SDD), providing a current pulse with amplitude proportional to the energy of the photon for any detected X-ray photon.

The X-ray beam collimation was 1 mm, and the acquisition time was 300 s. The spectra were analyzed with Pymca 5.9.2 [20]. Principal component analysis (PCA) was applied to the spectral data to identify patterns and groupings among the samples. The PCA was performed using R software (version 4.4.2, CRAN), a comprehensive statistical tool widely used for data analysis.

### Micro-Raman spectroscopy

Micro-Raman analyses were conducted at room temperature using an inVia™ confocal Raman spectrometer (Renishaw) with a 250-mm focal length. The setup included an 1800 l/mm holographic diffraction grating and a Peltier-cooled CCD detector. Two excitation laser wavelengths were employed: a continuous-wave Nd (Renishaw) diode-pumped solid-state laser with a wavelength of 532.1 nm (green laser) and an output power of 50 mW, and a Renishaw HeNe laser with a wavelength of 632.8 nm (red laser) and output power of 17.5 mW. The laser beams were focused on the samples using a 50 × short working-distance N-Plan objective (N.A. = 0.75, Leica Microsystems).

For measurements with the 633-nm (red) laser, the spectral range of 100–2000 cm⁻^1^ was recorded, with the laser beam focused to a 1-μm spot diameter using a 50 × objective. The spectra were collected with the laser power set to 1% of the nominal value. Similarly, when using the 532-nm (green) laser, the spectral range of 100–1850 cm⁻^1^ was acquired. The beam, focused to a spot size of approximately 0.9 μm through the same 50 × objective, also operated at 1% of the nominal laser power for spectral acquisition. For measurements with the 633-nm (red) laser, the spectral range of 100–2000 cm^−1^ was recorded, with the laser beam focused to a 1-μm spot diameter using the 50 × objective. The spectra were collected with the laser power set to 1% of the nominal value. Similarly, when using the 532-nm (green) laser, the response in the spectral range of 100–1850 cm^−1^ was acquired.

### X-ray powder diffraction

The main purpose of the XRPD measurements was to achieve information on the crystal structure of the pigments. For the XRPD analysis, a Bruker D8 ADVANCE diffractometer equipped with a Mo tube (Kα radiation, λ = 0.71 Å) was used. The instrument operated at a current of 30 mA and a voltage of 40 kV. Data were collected over a 2θ range of 5 to 30°, with a step size of 0.02° and an acquisition time of 3 s per step. These parameters were selected to optimize the resolution and ensure accurate phase identification of the blue pigments present in the samples.

## Results

### X-ray fluorescence spectroscopy

**Fig. 2:**
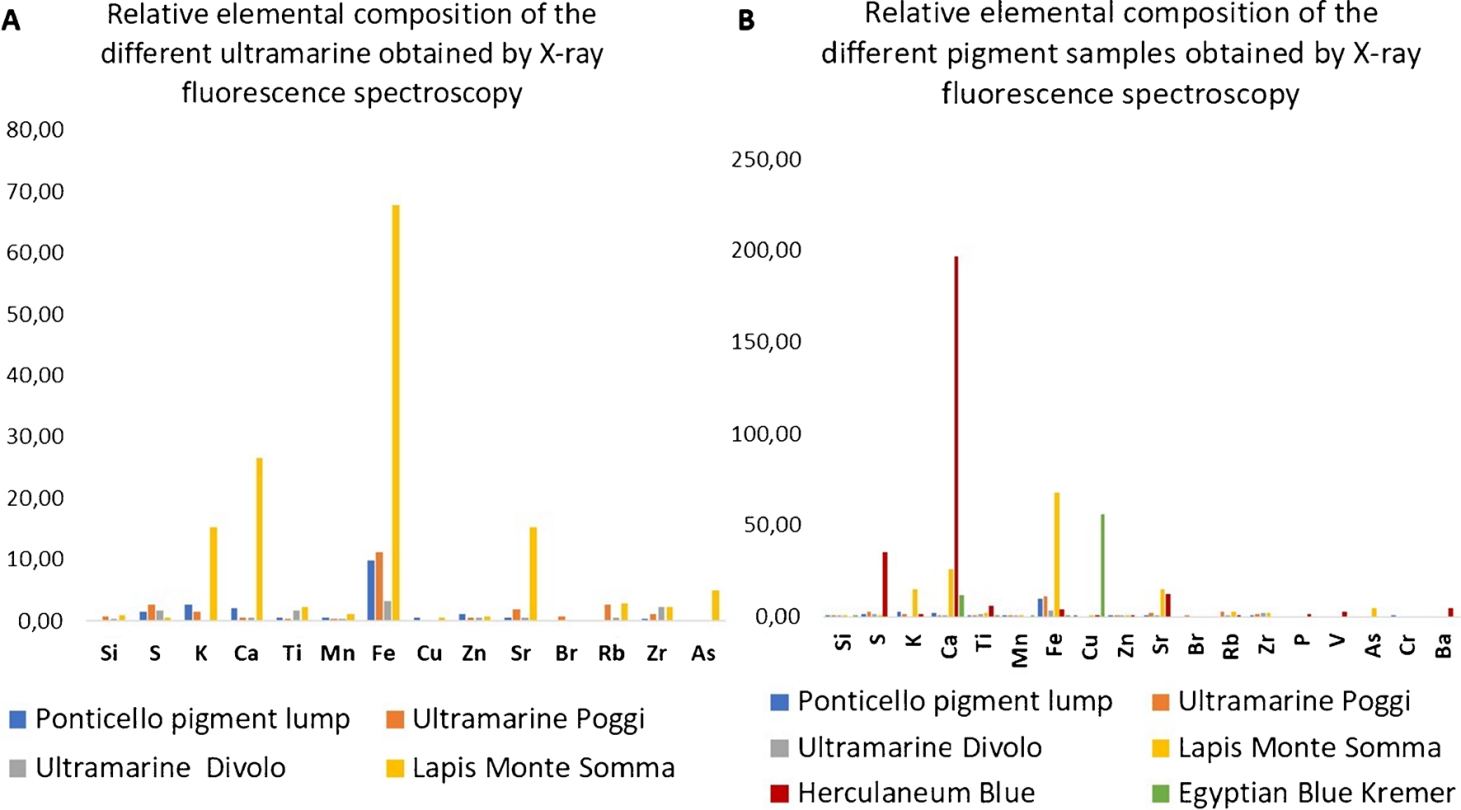
**A** Relative elemental composition of the different ultramarine obtained by ED-XRF spectroscopy. **B** Relative elemental composition of the different pigment samples obtained by ED-XRF spectroscopy

**Fig. 3:**
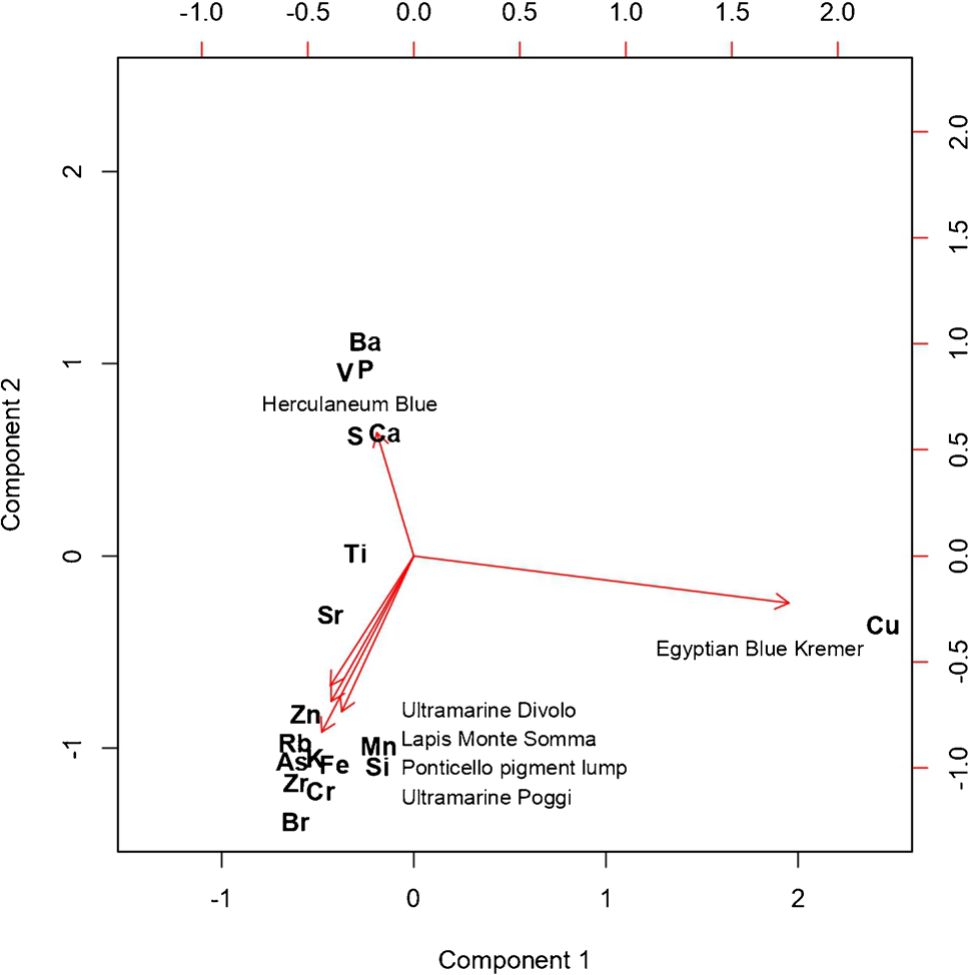
PCA of ED-XRF data comparing chemical composition of Ponticello pigment lump with other synthetic pigments such as Egyptian blue, Herculaneum blue, and various ultramarine pigments. The results indicate a significant variance between the pigments, with Egyptian blue showing the most distinct chemical composition, while the ultramarine pigments cluster closely, reflecting their chemical similarity

The ED-XRF analyses of these pigments are reported in Table S1 of the Supplementary Information. The synthetic commercial ultramarine blues (Poggi and Divolo) as well as pigment lump from Ponticello contain Fe and other impurities such as Zn, Mn, Br, Cr, and Zr which are documented both in the mineral and the synthetic forms, besides Si, K, and S, which are expected in ultramarine (lazurite) (Fig. 2a). Lapis lazuli from Monte Somma is distinguished by the presence of higher concentrations of elements including Fe, Ca, K, As, and Sr due to the presence of other minerals [21, 22]. In general, the synthetic samples of ultramarine show a higher concentration of S. Noticeable differences are also found in the content of Ca which is lower for the synthetic sample whereas the Ca content in natural samples is larger and clearly related to the quality, according to the literature [23]. No clear differences could be found in the content of Si. Commercial Herculaneum blue is associated with a higher concentration of Ca, S, and Ba due to the presence of gypsum (CaSO_4_·2H_2_O) and barite (BaSO_4_). Chalk and gypsum show Ca that is associated with its usual impurities Sr and Ti. In particular, Sr in gypsum is an impurity due to the presence of celestine (SrSO_4_). Traces of V and P are also detected (Fig. 2b). Egyptian blue (Kremer), a copper calcium silicate, is characterized by a high content of Cu, this element being essential for imparting its characteristic blue color. The six blue pigments are grouped based on their main characteristics ED-XRF lines in the PCA plot referring to the elemental composition of different blue pigments (Fig. 3). There are three distinct types of pigments: Herculaneum blue, ultramarine, and Egyptian blue. In the graph, each point represents a pigment sample. The principal axes (PC1 and PC2) represent the main directions of variation in the data. As shown in the plot, the synthetic commercial ultramarine blues (Poggi and Divolo) are positioned very close to each other and to the pigment lump from Ponticello. This indicates that they have a very similar chemical composition. The distinct position of Egyptian blue in the graph along the axis pointing toward Cu reflects its unique composition.

### Micro*-*Raman spectroscopy

**Fig. 4:**
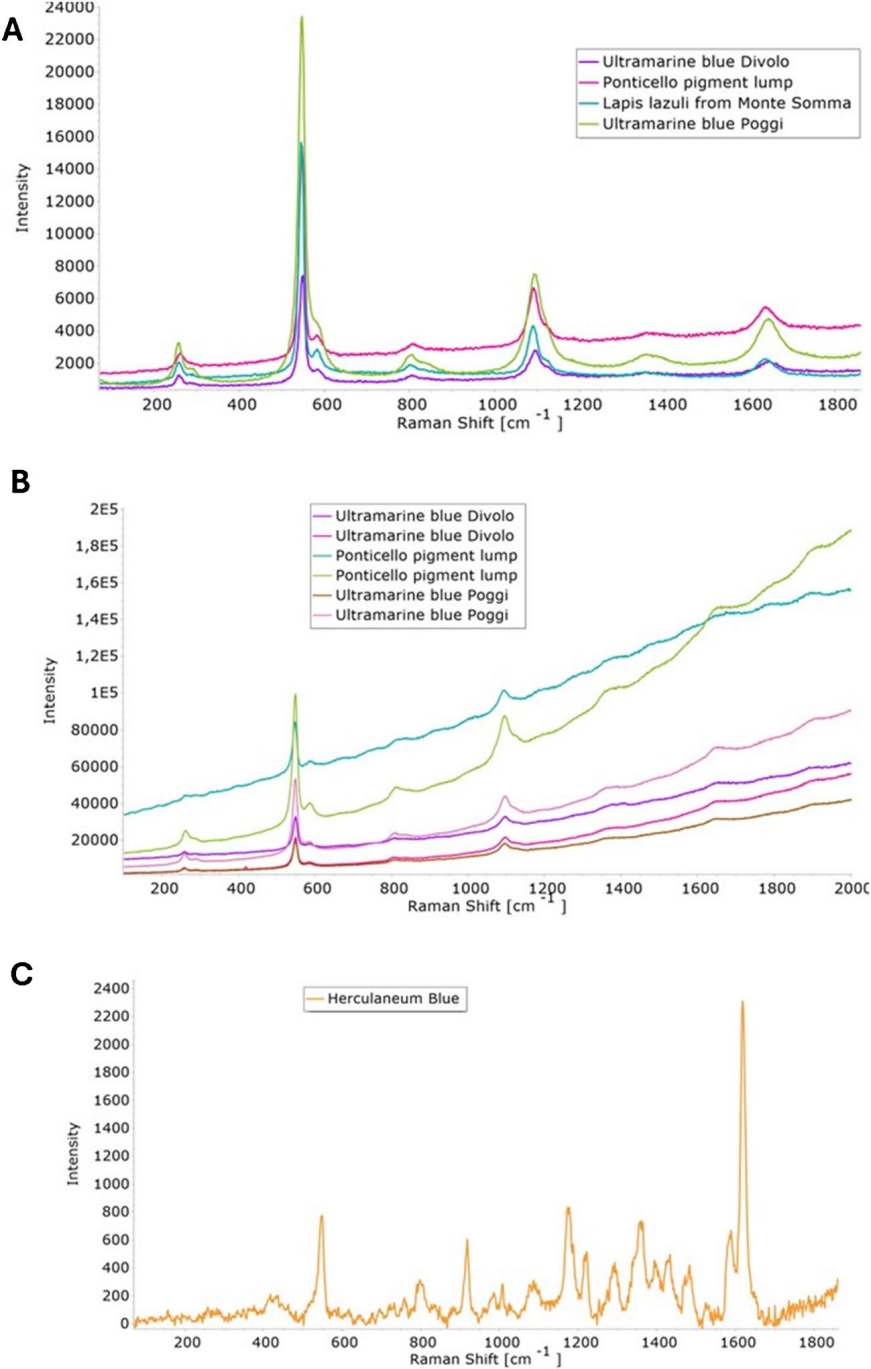
Micro-Raman spectra of Ponticello lump, synthetic ultramarine samples, and lapis lazuli from Monte Somma with **A** 532 nm laser excitation and **B** 632 nm laser excitation. In **B**, for each pigment, two different points of analysis have been considered. **C** Micro-Raman spectra of Herculaneum pigment with 532 nm laser excitation

In Fig. 4a, it is displayed the Raman spectra obtained with a laser having an excitation wavelength of 532 nm. The symmetric stretching vibration (ν1) of S_3_^−^ radicals at 548 cm^−1^ is prominently visible across all spectra of natural and artificial ultramarine pigments, with the Poggi pigment showing a particularly intense peak. The Ponticello pigment lump presents a peak with the same intensity as the Monte Somma lapis lazuli pigment, whereas the Divolo pigment exhibits a less intense peak. Additionally, the bending vibration (ν2) typical of S_3_^−^ at 257 cm^−1^ and the first overtone of the stretching vibration (1ν1) at 1095 cm^−1^ are also observable. The figure clearly indicates that the Raman spectra of the analyzed samples display similar chemical compositions and molecular conformations. However, an additional shoulder at 585 cm^−1^, typically associated with either the symmetric stretching of the S_2_^−^ radical or the asymmetric stretching of S_3_^−^, is more prominent in the Ponticello pigment lump and the Monte Somma lapis lazuli pigment, a feature that differentiates them from the artificial pigments. No differences in peak identification are detected when using a green or red laser for excitation (with wavelength range comprised between 532 and 633 nm). It is observed a broad background in the Raman spectra obtained with the red laser (Fig. 4b).

The spectrum in Fig. 4c, referring to the Raman signal of the Herculaneum pigment, reveals a mixture of different pigments. Specifically, it shows a peak at 550 cm^−1^, attributed to S_3_^−^ radicals in ultramarine, along with two strong bands at 1008 and 1088 cm^−1^, which are, in turn, characteristic of gypsum (CaSO_4_·2H_2_O) and calcite (CaCO_3_), respectively. Additionally, the spectrum includes signals from barite (BaSO_4_), with peaks around 463 and 988 cm^−1^, as well as some synthetic organic pigments featuring prominent Raman modes at 1591 and 1619 cm^−1^ [24]. These modes are associated with the class of monoazo pigments, but a comparison with Raman spectra literature did not permit the complete and unambiguous identification of these organic dyes.

### X-ray powder diffraction

**Fig. 5:**
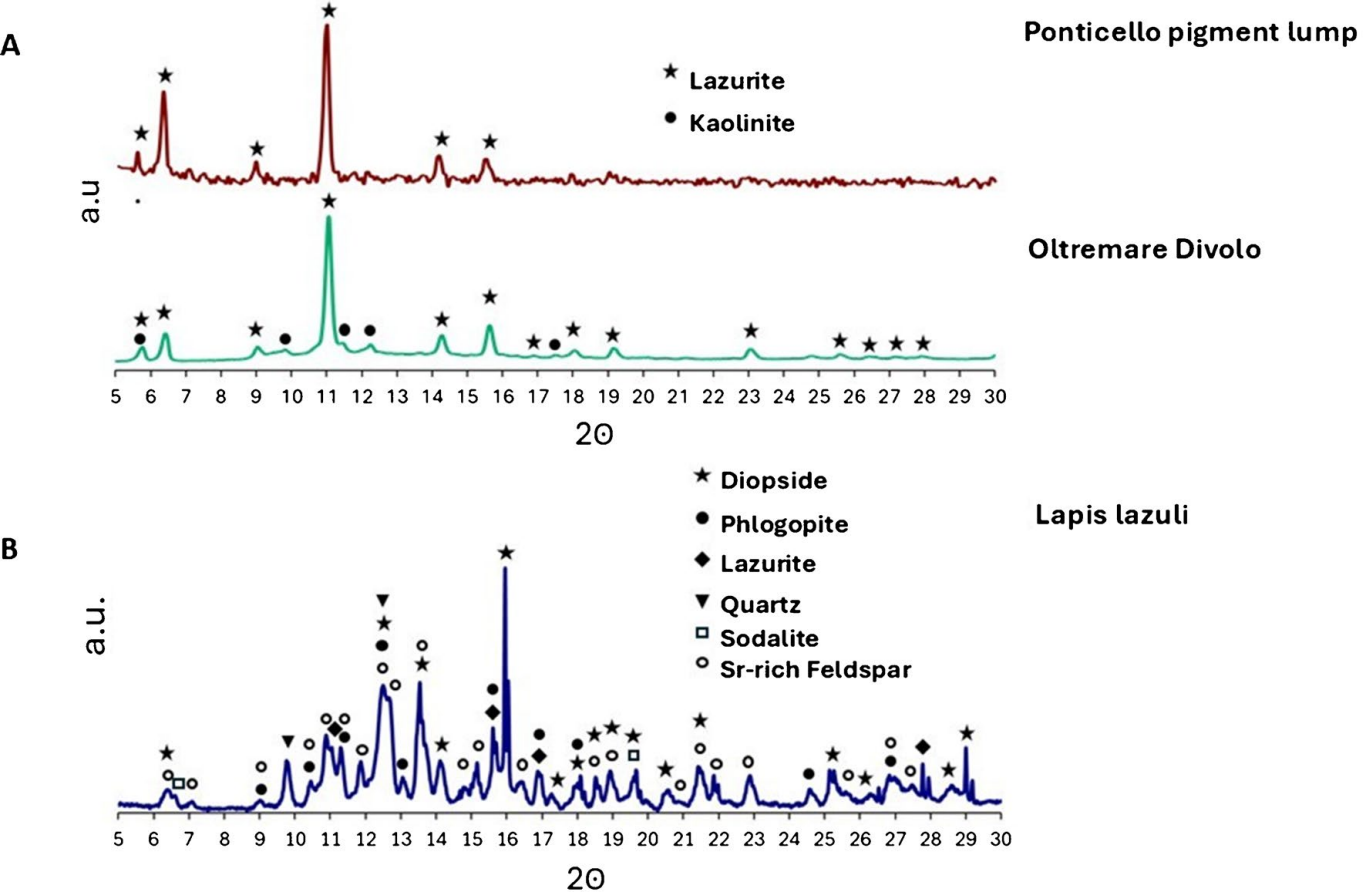
**A** X-ray diffractograms of Ponticello pigment lump and synthetic ultramarine from Divolo. **B** X-ray diffractogram of a natural lapis lazuli from Monte Somma, showing the presence of lazurite, diopside, phlogopite, sodalite, quartz, and Sr-rich feldspar

The Ponticello sample (Fig. 5a, in red) appears to be entirely crystalline and consists mostly of pure lazurite. Not all peaks of lazurite reference are visible, and some minor peaks, in particular at higher angles, appear obscured by noise, which is probably due to the small amount of available sample, a condition that limits the detection of signals from certain crystalline planes. Lazurite is a tectosilicate mineral composed of a mixture of sodium, and calcium sulfide, sulfate, hydroxide, and chloride, with a cubic crystal structure. A comparison of this sample with the synthetic Divolo pigment (green) is presented in Fig. 5a. While the Ponticello sample showed a pure lazurite crystalline composition, the diffractogram collected from a commercial reference sample (green curve) clearly shows lazurite and kaolinite as the major phases. Lazurite is the dominant phase, with kaolinite appearing less prominently. Previous studies also confirm that kaolinite is a common phase alongside lazurite in synthetic samples [25–27].

The sample of lapis lazuli from Monte Somma is particularly challenging to analyze with the XRD technique (Fig. 5b). The sample presents a complex matrix, typical of rocks, which are composed of intricate mixtures of mineral phases. The primary phases identified in this natural lapis lazuli sample include lazurite [Na_6_Ca_2_(Al_6_Si_6_O_24_)(SO_4_,S,S_2_,S_3_,Cl,OH)], sodalite [Na_8_(Al_6_Si_6_O_24_)Cl_2_], diopside (CaMgSi_2_O_6_), phlogopite (KMg_3_(Si_3_Al)O_10_(F,OH)_2_), quartz (SiO_2_), and Sr-rich feldspars.

## Discussion

The analyses conducted via ED-XRF, micro-Raman, and XRD provide a clear picture of the similarities and differences in the chemical and structural properties of the various blue pigments studied, allowing a thorough comparison with previous studies [28–32]. ED-XRF analysis, combined with PCA, revealed a distinct grouping of the pigments based on their chemical composition. Synthetic ultramarine pigments, such as Poggi and Divolo, cluster closely with the Ponticello pigment lump reflecting a similar composition dominated by Si, K, and S**—**elements typical of lazurite. Egyptian blue separates clearly due to its high Cu content, essential for its color. In contrast, natural lapis lazuli from Monte Somma exhibits a more complex composition, with elevated concentrations of Fe, Ca, K, and Sr, due to associated minerals. The natural pigment’s heterogeneous composition, as confirmed by ED-XRF, aligns with previous findings [28, 32], showing calcium associated with calcite and wollastonite. Calcium and magnesium are key markers of accessory minerals such as diopside and forsterite, frequently linked to natural pigments [29].

Raman spectroscopy identified the symmetric stretching peak of the S_3_^−^ radical anion at 545–548 cm^−1^ in all analyzed samples, a characteristic of both natural and synthetic ultramarine, as previously reported [28, 30, 31]. However, the Ponticello sample exhibits an additional shoulder at 584–586 cm^−1^, attributable to S_2_^−^ radical anion. This feature, absent in synthetic pigments, is frequently associated with structural impurities of a more complex mineralogical composition in natural pigments, as also highlighted in ref. 32. Unexpectedly, Raman peaks at 635 cm^−1^ and 970 cm^−1^, which are associated to wollastonite inclusions (CaSiO_3_), a mineral commonly found in natural pigments [28], are not detected in the spectrum of Ponticello pigment. Differences in the intensity of the main peak among the synthetic samples (Poggi and Divolo) suggest that the synthetic variants may have different minor minerals while sharing the same basic component.

The XRPD analysis provides a complementary view, highlighting differences in the crystalline structure of the pigments. The Ponticello pigment lump is almost entirely composed of pure lazurite, with no detectable kaolinite, unlike the commercial Divolo pigment. Synthetic ultramarine is produced through industrial processes involving kaolin dehydration at 550 °C, followed by sulfurization and reduction–oxidation steps at varying temperatures. These controlled conditions yield pure lazurite and a consistent blue hue. In contrast, the Monte Somma natural lapis lazuli displays structural complexity, with several minerals, e.g., lazurite, sodalite, diopside, phlogopite, and quartz. The heterogeneity of the natural sample complicates precise diffraction analysis, as overlapping phases obscure clear interpretation. The difference observed in the XRD analysis is because the lump from Ponticello is a purified lazurite, while the Monte Somma sample is raw powdered lapis lazuli, a natural rock containing multiple phases and minerals such as calcite and pyrite. Historical pigment purification techniques, like those described by Cennino Cennini [33], involved processes such as grinding lapis lazuli, binding it with waxes and resins, and repeatedly washing it in alkaline solutions. This method selectively extracted lazurite particles while removing non-blue impurities, resulting in a high-purity pigment suitable for artistic use. This explains why the Ponticello lump contains only lazurite, in contrast to the unprocessed Monte Somma sample that retains its natural mineral inclusions.

## Conclusions

In summary, the analyses conducted using ED-XRF spectroscopy, micro-Raman spectroscopy, and XRPD have convincingly identified the blue pigment lump from Ponticello as natural lazurite, ruling out the possibility that it is one of other blue pigments, such as Herculaneum blue or Egyptian blue that were commonly used in the antiquity. This research is particularly significant due to the rarity of ultramarine in Roman wall paintings.

As a precious material, the discovery of ultramarine in Roman artifacts is an exceptional find in itself. Previous FORS colorimetric analyses have already indicated the presence of ultramarine in the murals of the Volsinii forum latrina (forica), suggesting a sophisticated use of various blue pigments given the presence of various shades of blue in the forica mural, an information that stimulates further exploration. Through this investigation, the study contributes to shed light on the trade routes, material exchanges, and artistic practices of Roman times, providing a better understanding of the cultural and economic interactions during that period. The discovery of lazurite at Ponticello, a site recognized as a rural settlement during Roman times, not only provides clues about the pigments used in the frescoes located in public areas but also emphasizes the intricate trade networks that existed since ultramarine was traditionally sourced from distant regions like Afghanistan. However, the possibility that the pigment may have originated form closer, less-explored sources, such as Monte Somma, should not be ruled out. The presence of such a luxurious pigment suggests the elevated artistic standards and substantial economic resources. Furthermore, this research enhances our understanding of both natural and synthetic pigments through a multi-analytical approach. Notably, the Raman spectra reveal a distinct shoulder at 585 cm^−1^, linked to the S_2_^−^ and S_3_^−^ radical anions, which is characteristic of natural ultramarine. Additionally, XRPD analysis shows the presence of kaolinite in the blue pigment from Divolo, which was utilized in the industrial production of synthetic ultramarine. These insights contribute significantly to the differentiation and characterization of historical pigments.

## Supplementary Information

Below is the link to the electronic supplementary material.

Supplementary file1 (PDF 1078 KB)

## Data Availability

Data will be made available on request.
