# Supplementary material for: Characterization of ultramarine blue in Roman wall paintings: case study from Volsinii (Bolsena, Italy)
Source: Anal Bioanal Chem. 2025 Feb 8;417(8):1557–66. doi: 10.1007/s00216-025-05745-y (PMC11876258; doi:10.1007/s00216-025-05745-y)
Supplement: Supplementary file 1 — Supplementary file1 (PDF 1078 KB) [file 216_2025_5745_MOESM1_ESM.pdf]

*Table S1: XRF analyses of pigments, normalized by the duration of signal recording*

| Element | | Ponticello lump | Ultramarine Poggi | Ultramarine Di Volo | Lapis from Monte Somma | Herculaneum Blue | Egyptian Blue |
|---|---|---|---|---|---|---|---|
| **Si** | K | 0.23 | 0.74 | 0.40 | 0.91 | - | 0.36 |
| **S** | K | 1.56 | 2.75 | 1.74 | 0.58 | 35.31 | - |
| **K** | K | 2.64 | 1.62 | 0.07 | 15.27 | 1.35 | - |
| **Ca** | K | 2.09 | 0.55 | 0.47 | 26.58 | 197.30 | 11.86 |
| **Ti** | K | 0.58 | 0.29 | 1.68 | 2.28 | 5.89 | 0.31 |
| **Mn** | K | 0.50 | 0.29 | 0.32 | 1.15 | 0.49 | 0.37 |
| **Fe** | K | 9.97 | 11.21 | 3.34 | 67.79 | 4.08 | 1.08 |
| **Cu** | K | 0.50 | - | - | 0.56 | 1.12 | 56.14 |
| **Zn** | K | 1.06 | 0.58 | 0.56 | 0.79 | 0.53 | - |
| **Sr** | K | 0.56 | 1.89 | 0.62 | 15.35 | 12.46 | - |
| **Br** | K | - | 0.83 | - | - | - | - |
| **Rb** | K | - | 2.62 | 0.52 | 2.87 | 0.70 | - |
| **Zr** | K | - | 1.22 | 2.31 | 2.30 | - | - |
| **P** | K | - | - | - | - | 1.27 | - |
| **V** | K | - | - | - | - | 2.75 | - |
| **Nb** | K | - | - | - | - | 0.45 | - |
| **As** | K | - | - | - | 5.03 | - | - |

For Peer Review
